# Gender-and lesion number-dependent difference in “atherogenic index of plasma” in Chinese people with coronary heart disease

**DOI:** 10.1038/s41598-017-13267-6

**Published:** 2017-10-16

**Authors:** Wei Ni, Zhenyu Zhou, Tao Liu, Haoyu Wang, Jianping Deng, Xiaoyan Liu, Guoqiang Xing

**Affiliations:** 1 Department of Cardiology, Central Hospital of Nanchong, The Second Clinical School of North Sichuan Medical College, Nanchong, China; 2 Department of Cardiology, The first affiliated hospital of chongqing medical university, Chongqing, China; 3 Department of Geriatrics, Central Hospital of Nanchong, The Second Clinical School of North Sichuan Medical College, Nanchong, China; 4 Department of Imaging & Imaging Institute of Rehabilitation and Development of Brain Function, The Second Clinical School of North Sichuan Medical College, Nanchong, China

## Abstract

Few studies has investigated the interrelationship between Atherogenic index of plasma (AIP) and coronary heart disease (CHD) especially in Asians. AIP is the logarithmically transformed ratio of triglyceride (TG) to high-density lipoprotein cholesterol (HDL-C), and is thought to be associated with arteriosclerosis, hypertension, diabetes and cardiovascular diseases. Of the 463 patients from Central Hospital of Nanchong in 2011–2014 diagnosed with angiograms, 229 CHD (>50% stenosis in one or more arteries) and the rest 234 were the controls (maximum stenosis < 10% in any artery) according to the world health organization (who) diagnostic criteria. The multiple regression analysis showed that AIP was independently associated with CHD in men (odds ratio (OR) = 4.44, 95%CI 1.62–12.21, P = 0.004) after adjusting for age, body mass index (BMI), fasting blood glucose(FBG), homocysteine (Hcy), and smoking, but not in women (OR = 0.47, 95%CI 0.11–2.08, P = 0.318). Subgroup analysis showed that the significant difference in AIP between the CHD and the controls only exists in patients with multi-vessel lesions but not in those with single-vessel lesion. Further large-scale studies with balanced sex ratio and vessel lesion numbers should verify the present findings.

## Introduction

According to China’s Ministry of Health data, nearly 3.5 million people die each year from cardiovascular disease (CVD) in China and CVD is the main cause of death in Chinese population^1,2^. Therefore there is an urgent need to make early diagnosis and prevention measures to control of coronary heart disease (CHD).

Atherogenic index of plasma (AIP) is a useful independent predictor of all-cause mortality and cardiovascular events^3,4^. It had been designated as a surrogate of small low-density lipoprotein particle size^5,6^, and is associated with hypertension, diabetes, metabolic syndrome, and the risk of cardiovascular events^7–10^. A 7.8-year follow-up study of 2,676 middle-aged adults showed that AIP is a reliable biomarker for predicting diabetes and hypertension^11^. A cross-sectional study in Nigeria showed that significantly increased AIP was a useful predictor of cardiovascular risk among postmenopausal women^10^. Significantly increased AIP and intima-media thickness of the carotid artery were found in subclinical atherosclerosis in patients on maintenance hemodialysis in Turkey^12^. Similar findings of AIP as useful tool for the diagnosis and prognosis of CVD was reported in Morocco population^13^. However, there have been no reports regarding the relationship between AIP, CHD, and severity of coronary artery stenosis confirmed by coronary angiography.

To define the relationship between AIP and CHD, this study was designed to determine the association between AIP, CHD, and the severity of coronary artery lesions in a Chinese population.

## Methods

### Study population and diagnosis

This study was approved by the Ethics Committee, Central Hospital of Nanchong. Clinical trial registration number: ChiCTR-RRC-16008502 (http://www.chictr.org/en/). This was a retrospective study, and informed consent could not be obtained from each patient. Instead of obtaining informed consent from each patient, we posted a notice about the study design and contact information at a public location in our hospital. All experiments in this study were performed in accordance with the relevant guidelines and regulations. The participants were consenting individuals undergoing coronary angiography at the Central Hospital of Nanchong from 2011 to 2014, and were at least 18 years of age. All subjects were without a history of prior myocardial infarction or revascularization. The indications for angiography include exercise-induced chest pain and atypical chest pain. Coronary angiograms were obtained by using standard techniques. The angiograms were defined with respect to the number of vessels involved (0, 1, 2, or 3). A total of 229 patients met the diagnostic criteria of CHD set by the World Health Organization, i.e. an individual with CHD should have at least one coronary stenosis ≥50%. Those participants with a maximum stenosis of < 10% in any artery were included as the controls. Major exclusion criteria included: 1) a history of coagulation disorders; 2) familial hypercholesterolemia; 3) New York Heart Association Class III and Class IV heart failure; 4) ongoing systemic inflammatory diseases; 5) renal or hepatic dysfunction; 6) significant valvular disease, myocarditis, cardiomyopathies, and malignancy; 7) patient’s unwillingness to participate in the survey. Hypertension was defined as systolic blood pressure greater than 140 mmHg, diastolic blood pressure greater than 90 mmHg, or a history of hypertension. Diabetes was defined according to the plasma glucose criteria of the World Health Organi-zation: fasting plasma glucose greater than 7.8 mmol/l and/or 2-hour postload glucose level greater than 11.1 mmol/l. Smoking were defined as adults aged ≥18 years who reported having smoked ≥100 cigarettes and currently smoked every day or some days.

### Data collection

Detailed demographic characteristics of each study object were recorded. Baseline data were ascertained by trained research staff according to standard operating procedures. Fasting venous blood was drawn from the forearm of each participant. Fasting blood glucose (FBG), triglyceride (TG), total cholesterol (TC), low-density lipoprotein (LDL), high-density lipoprotein (HDL), C-reactive protein (CRP), and homocysteine (Hcy) were measured using a Hitachi 7020 automatic blood analyzer (Hitachi, Tokyo, Japan). AIP was calculated as the logarithmically transformed ratio of TG to HDL-C, i.e., [log(*TG*/*HDL-C*)], measured in mmol/L.

### Statistical analysis

All data were double-entered before being exported to tab-delimited text files. All analyses were performed by using R (http://www.R-project.org) and EmpowerStats software (www.empowerstats.com, X&Y solutions, Inc. Boston, MA). Mean and proportion of the CHD group and the control group were calculated for the demographic characteristics and the blood analysis. The differences in population characteristics were compared using the Student’s *t*-test or the chi-square test. Associations between risk factors and CHD severity were expressed as the odds ratio (OR). Multiple regression analyses were used to evaluate the relationship between AIP and CHD after adjusting for various variables. Differences were considered to be significant at *P* < 0.05.

## Results

Table 1 shows the demographics of the CHD group and the control group. No significant differences in mean age between the CHD group and the control group. The CHD group had greater values than the control group in TG, FBG, Hcy, AIP levels and smoking. No significant difference found in BMI, TC, LDL, HDL, CRP, and the proportion of hypertension and diabetes between the CHD and control groups. Univariate analysis showed that CHD was significantly associated with male sex, TG, AIP, FBG, Hcy, and smoking. Blood levels of TC, LDL, HDL, CRP were not significantly different between the two groups. It is noted, however, that the distribution of hypertension and diabetes, two common risk factors of CHD, were not statistically different between the CHD and control groups **(**Table 2 **)**. Therefore, male sex, enhanced values of TG, AIP, FBG, Hcy, and smoking were found to be the risk factors for CHD in this study.

**Table 1:** Demographic and clinical characteristics of study subjects.

Male, N(%)	Control (N = 234)	CHD group (N = 229)	P value
	135 (57.7)	171 (74.7)	<0.001
Age(y)	65.7 ± 9.4	64.9 ± 10.0	0.365
BMI(kg/m^2^)	23.9 ± 3.1	23.8 ± 3.1	0.856
TG(mmol/L)	1.3 [0.5–9.7]	1.6 [0.5–11.2]	0.002
TC(mmol/L)	4.8 ± 1.0	4.8 ± 1.2	0.960
LDL(mmol/L)	3.0 ± 0.9	2.8 ± 1.0	0.053
HDL(mmol/L)	1.1 ± 0.3	1.1 ± 0.4	0.248
FBG(mmol/L)	6.7 ± 2.3	8.2 ± 4.8	<0.001
CRP(mg/L)	2.3 [0.1–278.3]	2.8 [0.1–317.2]	0.924
Hcy(μmol/L)	13.4 ± 6.4	15.0 ± 7.0	0.010
AIP	0.1 [−0.5–1.0]	0.2 [−0.6–1.0]	0.006
Smoking, N(%)	48 (20.5)	91 (40.1)	<0.001
Hypertension, N(%)	110 (47.0)	120 (52.9)	0.209
Diabetes, N(%)	35 (15.0)	48 (21.1)	0.084
Coronary artery lesions, N(%)			< 0.001
Normal	234 (100.0)	0	
Single-vessel lesion	0	98 (42.8)	
Multi-vessel lesions	0	131 (57.2)	

BMI = body mass index, TG = triglyceride, TC = total cholesterol, LDL = low-density lipoprotein, HDL = high-density lipoprotein, FBG = fasting blood glucose, CRP = C-reactive protein, Hcy = homocysteine, AIP = atherogenic index of plasma.

**Table 2:** Relationship of AIP with CHD by univariate analysis.

	N	Odd ratio (95% CI)	*P* value
Age	461	0.99 (0.97, 1.01)	0.365
Gender	463		
Female		Ref	
Male		2.16 (1.46, 3.21)	<0.001
BMI	420	0.99 (0.93, 1.06)	0.855
TG	458	1.32 (1.10, 1.58)	0.003
TC	458	0.99 (0.84, 1.17)	0.96
LDL	453	0.82(0.67, 1.00)	0.054
HDL	457	1.36 (0.80, 2.31)	0.253
AIP	457	2.52(1.29, 4.94)	0.007
FBG	461	1.15 (1.07, 1.23)	<0.001
CRP	350	1.00 (0.99, 1.01)	0.924
Hcy	460	1.04 (1.01, 1.07)	0.012
Smoking	461		
No		Ref	
Yes		2.59 (1.71, 3.92)	< 0.001
Diabetes	461		
No		Ref	
Yes		1.52 (0.94, 2.46)	0.085
Hypertension	461		
No		Ref	
Yes		1.26 (0.88, 1.82)	0.209

BMI = body mass index, TG = triglyceride, TC = total cholesterol, LDL = low-density lipoprotein, HDL = high-density lipoprotein, AIP = atherogenic index of plasma, FBG = fasting blood glucose, CRP = C-reactive protein, Hcy = homocysteine.

Multivariable logistic regression analysis showed that AIP was independently associated with CHD in men (OR = 4.90, 95%CI 2.11–11.38, *P* = 0.001) and this relationship remained significant after adjustment for age, BMI, FBG, Hcy, and smoking (OR = 4.44, 95%CI 1.62–12.21, *P* = 0.004). The results of the correlation analysis between the quartiles of AIP and CHD showed that Q4 of AIP was independently associated with CHD and the results of the trend test were also significant. However, these results were not found in women **(**Table 3 **)**.

**Table 3:** Relationship of AIP with CHD by multivariable logistic-regression model according to gender.

	**Model 1**				**Model 2**	
	N	Odd ratio (95% CI)	*P*	N	Odd ratio (95% CI)	*P* Value
**Female**	155			139		
**AIP**	155	0.75 (0.21, 2.60)	0.646	139	0.47 (0.11, 2.08)	0.318
**Quartiles of AIP**	155			139		
Q1		Ref			Ref	
Q2		1.60 (0.59, 4.29)	0.352		1.16 (0.38, 3.60)	0.795
Q3		1.92 (0.74, 4.99)	0.181		1.67 (0.55, 5.06)	0.366
Q4		0.85 (0.28, 2.60)	0.776		0.43 (0.11, 1.75)	0.239
**P for trend**	155	0.99 (0.72, 1.37)	0.966	139	0.87 (0.59, 1.29)	0.494
**Male**	302					
**AIP**	302	4.90 (2.11, 11.38)	0.001	273	4.44 (1.62, 12.21)	0.004
**Quartiles of AIP**	302			273		
Q1		Ref			Ref	
Q2		0.88 (0.46, 1.66)	0.683		0.862 (0.41, 1.81)	0.693
Q3		1.74 (0.89, 3.38)	0.100		1.51 (0.694 3.27)	0.300
Q4		2.18 (1.17, 4.07)	0.014		2.10 (1.00, 4.37)	0.049
**P for trend**	302	1.34 (1.10, 1.64)	0.004	273	1.32 (1.04, 1.67)	0.021

Model 1: Crude.

Model 2: Adjusted age, BMI, FBG, Hcy and Smoking.

AIP = atherogenic index of plasma.

**Figure 1:**
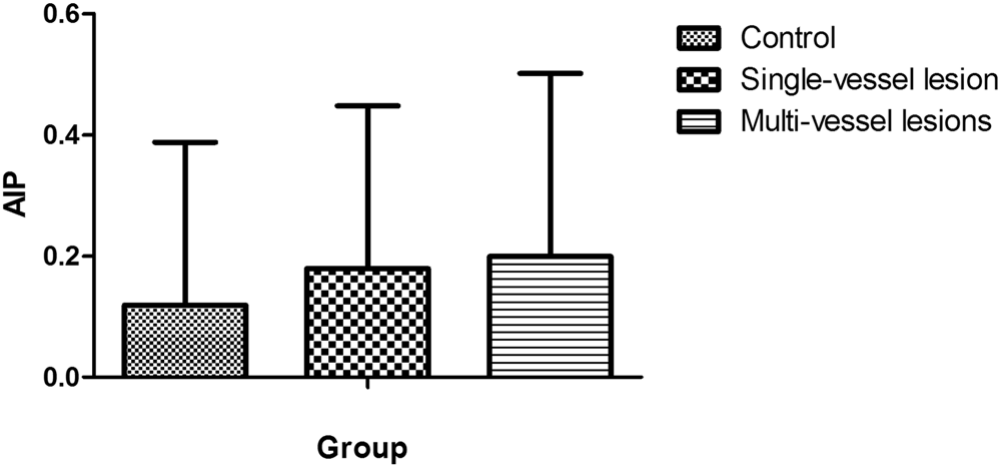
Analysis of variance between control, single-vessel lesion and multi-vessel lesions. The difference between multivessel lesions group and control group was statistically significant (P = 0.028), and there was no statistical significance between the other groups (P > 0.05).

Further variance analysis between AIP and the severity of coronary artery stenosis, significant difference was found between the multivessel lesions group and the control group (*P* = 0.028), but not in other groups (*P* > 0.05) **(**Fig. 1 **)**.

## Discussion

In the present study, we performed a cross-sectional study of 463 participants (306 men and 157 women) to investigate the association between AIP-which is the logarithmically transformed ratio of triglyceride to HDL-C and CHD, and the severity of coronary artery lesion. Univariate analysis and multivariate logistic regression analyses showed that AIP was independently associated with CHD in men but not in women. Further, within-CHD group analysis showed that AIP in the multivessel lesions group but not in the single-vessel lesion group was significantly higher than that in the control group. These results indicate that AIP is independently associated with CHD in male patients and is connected with multivessel lesions.

Dobiasova first proposed in 2001 that AIP is closely linked with the esterification rate of apoB-lipoprotein-depleted plasma and with the lipoprotein particle size, and thus could be used as a biomarker of plasma atherogenicity^6^. As far as we know, our study is the first to show that AIP is independently associated with CHD in males. We also found a relationship between AIP and multivessel lesions in Chinese CHD patients.

Previous studies have shown that increased AIP value is associated with increased risk of CHD. The normal AIP values in young and healthy women are below 0.11 while the AIP value in men and subjects with increased risk of CHD such as hypertension, diabetes mellitus, and dyslipidemia can reach up to 0.4^7^. According to the published epidemiological data, subjects of CHD risk can be further divided into three subgroups based on their AIP values: 1) low CHD risk: with the AIP values between –0.3 and 0.11 with; medium risk: with the AIP values between 0.11 to 0.21; and 3) high CHD risk: with the AIP values above 0.21 with^14^. In our study, the AIP values of the CHD group is 0.2 [−0.6–1.0]. Most of the participants of this study fell into the medium-high groups.

No significant difference in blood TC, HDL, and LDL were found between the CHD and control groups. AIP is a logarithmically transformed ratio of the molar concentrations of TG and HDL-C, and it has been designated as a surrogate of small LDL particle size because AIP may be more closely associated with the risk of CHD.

A cross-sectional study showed a high risk of cardiovascular events (AIP = 0.369 ± 0.04) in nonobese sedentary Nigerian men^15^. Another prospective study showed high AIP in both Turkish men and women as the strong risk factor for CHD^11^. Also, a US study of the Women’s Ischemia Syndrome Evaluation indicates that AIP was a significant independent predictor of all-cause mortality and cardiovascular events in women without prior myocardial infarction or coronary revascularization. In our present study, high AIP is associated with CHD in men but not found in women. These inconsistent results of gender difference in AIP may be due to differences in culture, race, diet, lifestyle, demographic characteristics and different inclusion criteria, and laboratory tests. While no ready explanation can be given yet, different postmenopausal and metabolic status could have also affected the AIP values. Nwagha *et al*.^10^ showed a AIP value of –0.17 before menopause that increased significantly to 0.15 in postmenopause Nigeria women. Essiarab noted that a mean AIP value of 0.21 in obese women without metabolic syndrome that was doubled in the presence of metabolic syndrome^13^. At the same time, it may be related to the fact that the number of the women participants was smaller and about half of the men in our study. Similarly, the significantly higher AIP values in the multivessel lesion group than in the control group, but not in the single-vessel lesion group could also be due to statistical bias because of the fewer single-vessel lesion cases.

As mentioned above, one limitation of our study is the relatively smaller sample size of female participants that may have caused a bias towards male findings. Also, our study population is from underdeveloped regions in Southwestern China, it is unknown yet whether such findings can be extended to other regions. Although increased AIP are reported to be associated with the risk of type 2 diabetes mellitus^8,9^, hypertension^11^, mood disorders^16^, human immunodeficiency virus infection^17^, schistosome infection^18^, hyperuricemia^19^, and preeclampsia^20^, the present study could only effectively evaluate the relationship between AIP and CHD.

Many studies revealed gender differences in the presentation, prevalence, and clinical outcomes of CHD^21–23^. Compared with females, ST-segment elevation myocardial infarction is more often diagnosed in men^24^. These findings indicate that gender may have an important influence on CHD. We agree with the research perspectives that an atherogenic LDL profile in postmenopausal women, comparable to that in men, may be related to both ageing and the menopause^25^. The use of statins increased among US adults with high coronary heart disease (CHD) risk after publication of the 2001 cholesterol treatment guidelines. Lisandro suggested that the preferential use of statins by individuals with high CHD risk in the contemporary era may induce a bias in analyses of the association between serum lipids and CHD. This bias may have important implications for future studies of lipids and CHD risk and for CHD risk prediction^26^.

In conclusion, increased AIP is independently associated with CHD in Chinese males and in people with multivessel lesions even after adjusting for traditional risk factors. Further studies are required to investigate the underlying mechanisms and the gender differences regarding the relationship between AIP and CHD.

## Electronic supplementary material

Supplementary Information

## References

[1] Hu SS (2012). Outline of the report on cardiovascular disease in China, 2010. Biomedical and environmental sciences: BES.

[2] Celermajer DS, Chow CK, Marijon E, Anstey NM, Woo KS (2012). Cardiovascular disease in the developing world: prevalences, patterns, and the potential of early disease detection. Journal of the American College of Cardiology.

[3] Bittner V (2009). The triglyceride/high-density lipoprotein cholesterol ratio predicts all-cause mortality in women with suspected myocardial ischemia: a report from the Women’s Ischemia Syndrome Evaluation (WISE). American heart journal.

[4] Wan K (2015). The association between triglyceride/high-density lipoprotein cholesterol ratio and all-cause mortality in acute coronary syndrome after coronary revascularization. PloS one.

[5] Dobiasova M, Frohlich J (2000). [The new atherogenic plasma index reflects the triglyceride and HDL-cholesterol ratio, the lipoprotein particle size and the cholesterol esterification rate: changes during lipanor therapy]. Vnitrni lekarstvi.

[6] Dobiasova M, Frohlich J (2001). The plasma parameter log (TG/HDL-C) as an atherogenic index: correlation with lipoprotein particle size and esterification rate in apoB-lipoprotein-depleted plasma (FER(HDL). Clinical biochemistry.

[7] Dobiásová M (2006). [AIP–atherogenic index of plasma as a significant predictor of cardiovascular risk: from research to practice]. Vnitřní Lékařství.

[8] Tan MH, Johns D, Glazer NB (2004). Pioglitazone reduces atherogenic index of plasma in patients with type 2 diabetes. Clinical chemistry.

[9] Zhu XW, Deng FY, Lei SF (2015). Meta-analysis of Atherogenic Index of Plasma and other lipid parameters in relation to risk of type 2 diabetes mellitus. Primary care diabetes.

[10] Nwagha UI, Ikekpeazu EJ, Ejezie FE, Neboh EE, Maduka IC (2010). Atherogenic index of plasma as useful predictor of cardiovascular risk among postmenopausal women in Enugu, Nigeria. African Health Sciences.

[11] Onat A, Can G, Kaya H, Hergenç G (2010). “Atherogenic index of plasma” (log10 triglyceride/high-density lipoprotein-cholesterol) predicts high blood pressure, diabetes, and vascular events. Journal of Clinical Lipidology.

[12] Yildiz G (2013). Evaluation of association between atherogenic index of plasma and intima-media thickness of the carotid artery for subclinic atherosclerosis in patients on maintenance hemodialysis. Hemodialysis International.

[13] Essiarab F, Taki H, Lebrazi H, Sabri M, Saïle R (2014). Usefulness of lipid ratios and atherogenic index of plasma in obese Moroccan women with or without metabolic syndrome. Ethnicity & Disease.

[14] Akbas EM (2014). *Association of uric acid*, *atherogenic index of plasma and al*buminuria in diabetes mellitus. International Journal of Clinical & Experimental Medicine.

[15] Ezeukwu AO, Agwubike EO (2013). Anthropometric measures of adiposity as correlates of atherogenic index of plasma in non-obese sedentary Nigerian males. Libyan Journal of Medicine.

[16] Nunes SO (2014). Atherogenic index of plasma and atherogenic coefficient are increased in major depression and bipolar disorder, especially when comorbid with tobacco use disorder. Journal of Affective Disorders.

[17] Onyedum CC, Young EE, Iroezindu MO, Chukwuka CJ, Nwagha UI (2014). Atherogenic index of plasma in highly active antiretroviral therapy-naïve patients with human immunodeficiency virus infection in Southeast Nigeria. Indian Journal of Endocrinology & Metabolism.

[18] Shen SW (2015). Potential long-term effects of previous schistosome infection may reduce the atherogenic index of plasma in Chinese men. International Journal for Parasitology.

[19] Baliarsingh S, Sharma N, Mukherjee R (2013). Serum uric acid: marker for atherosclerosis as it is positively associated with “atherogenic index of plasma”. Archives of Physiology & Biochemistry.

[20] Aragon-Charris J (2014). [Atherogenic index of plasma in patients with preeclampsia and in healthy pregnant women]. Medicina Clínica.

[21] Shaw LJ (2006). Insights from the NHLBI-Sponsored Women’s Ischemia Syndrome Evaluation (WISE) Study: Part I: gender differences in traditional and novel risk factors, symptom evaluation, and gender-optimized diagnostic strategies. Journal of the American College of Cardiology.

[22] Wake R, Takeuchi M, Yoshikawa J, Yoshiyama M (2007). Effects of gender on prognosis of patients with known or suspected coronary artery disease undergoing contrast-enhanced dobutamine stress echocardiography. Circulation Journal Official Journal of the Japanese Circulation Society.

[23] Sherazi, S. & Fisher, S. G. *Cardiovascular Disease in Women: Epidemiology of Cardiovascular Disease in Women- Sex Differences in Disease Incidence and Prevalence*. *Population Representation*, *Diversity*, *Disparities*. (Springer London, 2014).

[24] Barylski M, Mikhailidis DP, Ciebiada M, Rysz J, Banach M (2011). Gender differences in the treatment of ischemic heart disease. Current Pharmaceutical Design.

[25] Anagnostis P, Stevenson JC, Crook D, Johnston DG, Godsland IF (2016). Effects of gender, age and menopausal status on serum apolipoprotein concentrations. Clinical Endocrinology.

[26] Colantonio LD (2015). Association of Serum Lipids and Coronary Heart Disease in Contemporary Observational Studies. Circulation.

