# Supplementary material for: Gender-and lesion number-dependent difference in “atherogenic index of plasma” in Chinese people with coronary heart disease
Source: Sci Rep. 2017 Oct 16;7:13207. doi: 10.1038/s41598-017-13267-6 (PMC5643382; doi:10.1038/s41598-017-13267-6)
Supplement: Supplementary file 1 — Supplementary Information [file 41598_2017_13267_MOESM1_ESM.pdf]

Gender-and lesion number-dependent difference in “atherogenic index of plasma” in Chinese people with coronary heart disease(supplementary tables and figures)

Wei Ni[1], Zhenyu Zhou[1,2], Tao Liu[1*], Haoyu Wang[1], Jianping Deng[1], Xiaoyan Liu[3], Guoqiang Xing[4]

[1]Department of Cardiology, Central Hospital of Nanchong, The Second Clinical School of North Sichuan Medical College, Nanchong, China
[2]Department of Cardiology, The first affiliated hospital of chongqing medical university, Chongqing, China
[3]Department of Geriatrics, Central Hospital of Nanchong, The Second Clinical School of North Sichuan Medical College, Nanchong, China
[4]Department of Imaging & Imaging Institute of Rehabilitation and Development of Brain Function, The Second Clinical School of North Sichuan Medical College, Nanchong, China

[*]Corresponding author:
Tao Liu, MD
Department of Cardiology
Central Hospital of Nanchong
The Second Clinical School of North Sichuan Medical College
Nanchong, China
Tel: 86-13890856707
Fax: 86-0817-2223247
email nclt456@sina.com.

Running title:

Total words: 2,279 words (excluding references, tables, figures)
References: 26
Tables:3
Figures:1

Table1. Demographic and clinical characteristics of study subjects

	Control(N=234)	CHD group(N=229)	P value
Male, N(%)	135 (57.7)	171 (74.7)	<0.001
Age(y)	65.7± 9.4	64.9 ± 10.0	0.365
BMI(kg/m^2)	23.9 ± 3.1	23.8 ± 3.1	0.856
TG(mmol/L)	1.3 [0.5-9.7]	1.6 [0.5-11.2]	0.002
TC(mmol/L)	4.8± 1.0	4.8 ± 1.2	0.960
LDL(mmol/L)	3.0 ± 0.9	2.8 ± 1.0	0.053
HDL(mmol/L)	1.1± 0.3	1.1 ± 0.4	0.248
FBG(mmol/L)	6.7 ± 2.3	8.2 ± 4.8	<0.001
CRP(mg/L)	2.3 [0.1-278.3]	2.8 [0.1-317.2]	0.924
Hcy(μmol/L)	13.4 ± 6.4	15.0± 7.0	0.010
AIP	0.1 [-0.5-1.0]	0.2 [-0.6-1.0]	0.006
Smoking, N(%)	48 (20.5)	91 (40.1)	<0.001
Hypertension, N(%)	110 (47.0)	120 (52.9)	0.209
Diabetes, N(%)	35 (15.0)	48 (21.1)	0.084
Coronary artery lesions, N(%)			<0.001
Normal	234(100.0)	0	
Single-vessel lesion	0	98(42.8)	
Multi-vessel lesions	0	131(57.2)	

BMI = body mass index, TG = triglyceride, TC = total cholesterol, LDL = low-density lipoprotein, HDL = high-density lipoprotein, FBG = fasting blood glucose, CRP = C-reactive protein, Hcy = homocysteine, AIP = atherogenic index of plasma.

Table 2 Relationship of AIP with CHD by univariate analysis

	N	Odd ratio(95% CI)	*P* value
Age	461	0.99 (0.97, 1.01)	0.365
Gender	463		
Female		Ref	
Male		2.16 (1.46, 3.21)	<0.001
BMI	420	0.99 (0.93, 1.06)	0.855
TG	458	1.32 (1.10, 1.58)	0.003
TC	458	0.99 (0.84, 1.17)	0.96
LDL	453	0.82(0.67, 1.00)	0.054
HDL	457	1.36 (0.80, 2.31)	0.253
AIP	457	2.52(1.29, 4.94)	0.007
FBG	461	1.15 (1.07, 1.23)	<0.001
CRP	350	1.00 (0.99, 1.01)	0.924
Hcy	460	1.04 (1.01, 1.07)	0.012
Smoking	461		
No		Ref	
Yes		2.59 (1.71, 3.92)	<0.001
Diabetes	461		
No		Ref	
Yes		1.52 (0.94, 2.46)	0.085
Hypertension	461		
No		Ref	
Yes		1.26 (0.88, 1.82)	0.209

BMI = body mass index, TG = triglyceride, TC = total cholesterol, LDL = low-density lipoprotein, HDL = high-density lipoprotein, AIP = atherogenic index of plasma, FBG = fasting blood glucose, CRP = C-reactive protein, Hcy = homocysteine.

Table 3 Relationship of AIP with CHD by multivariable logistic-regression model according to gender

	Model 1			Model 2		
	N	Odd ratio(95% CI)	*P*	N	Odd ratio(95% CI)	*P* Value
**Female**	155			139		
**AIP**	155	0.75 (0.21, 2.60)	0.646	139	0.47 (0.11, 2.08)	0.318
**Quartiles of AIP**	155			139		
Q1		Ref			Ref	
Q2		1.60 (0.59, 4.29)	0.352		1.16 (0.38, 3.60)	0.795
Q3		1.92 (0.74, 4.99)	0.181		1.67 (0.55, 5.06)	0.366
Q4		0.85 (0.28, 2.60)	0.776		0.43 (0.11, 1.75)	0.239
**P for trend**	155	0.99 (0.72, 1.37)	0.966	139	0.87 (0.59, 1.29)	0.494
**Male**	302					
**AIP**	302	4.90 (2.11, 11.38)	0.001	273	4.44 (1.62, 12.21)	0.004
**Quartiles of AIP**	302			273		
Q1		Ref			Ref	
Q2		0.88 (0.46, 1.66)	0.683		0.862(0.41, 1.81)	0.693
Q3		1.74 (0.89, 3.38)	0.100		1.51 (0.694 3.27)	0.300
Q4		2.18 (1.17, 4.07)	0.014		2.10 (1.00, 4.37)	0.049
**P for trend**	302	1.34 (1.10, 1.64)	0.004	273	1.32 (1.04, 1.67)	0.021

Model 1: Crude

Model 2: Adjusted age, BMI, FBG, Hcy and Smoking

AIP = atherogenic index of plasma.

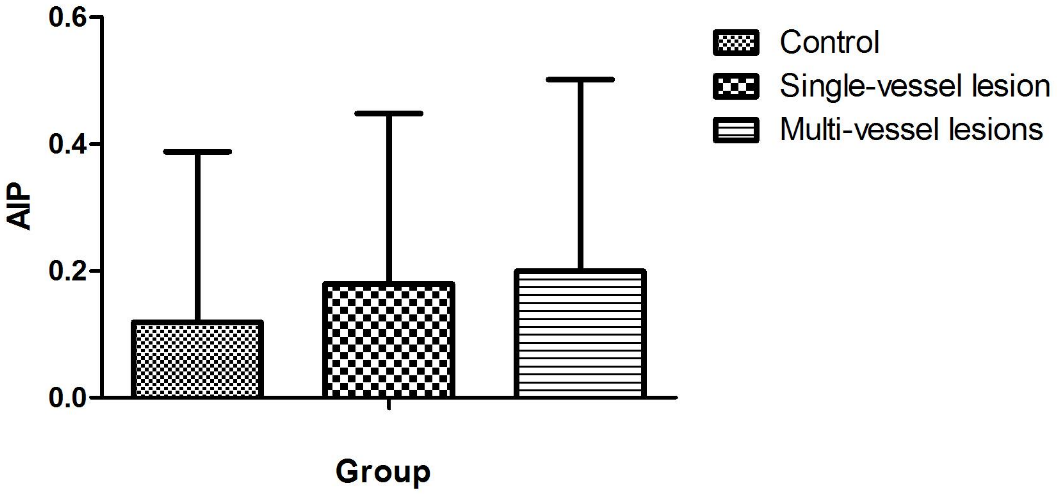

Fig 1. Analysis of variance between control, single-vessel lesion and multi-vessel lesions. The difference between multivessel lesions group and control group was statistically significant (P=0.028), and there was no statistical significance between the other groups(P>0.05).
